# The predictive capabilities of a novel cardiovascular magnetic resonance derived marker of cardiopulmonary reserve on established prognostic surrogate markers in patients with pulmonary vascular disease: results of a longitudinal pilot study

**DOI:** 10.1186/s12968-016-0316-4

**Published:** 2017-01-09

**Authors:** Timothy J. Baillie, Samuel Sidharta, Peter M. Steele, Stephen G. Worthley, Scott Willoughby, Karen Teo, Prashanthan Sanders, Stephen J. Nicholls, Matthew I. Worthley

**Affiliations:** 1 Cardiovascular Investigational Unit, Royal Adelaide Hospital, Adelaide, SA 5000 Australia; 2 University of Adelaide, Adelaide, Australia; 3 South Australian Health and Medical Research Institute, Adelaide, Australia

## Abstract

**Background:**

No unified method exists to effectively predict and monitor progression of pulmonary arterial hypertension (PAH). We assessed the longitudinal relationship between a novel marker of cardiopulmonary reserve and established prognostic surrogate markers in patients with pulmonary vascular disease.

**Methods and Results:**

Twenty participants with confirmed (*n* = 14) or at high risk (*n* = 6) for PAH underwent cardiovascular magnetic resonance (CMR) at baseline and after ~6 months of guideline-appropriate management. Ten PAH participants underwent RHC within 48 h of each CMR. RHC (mean pulmonary arterial pressure, mPAP; pulmonary vascular resistance index, PVRI; cardiac index, CI) and phase-contrast CMR (mean pulmonary arterial blood flow velocity, meanPAvel) measurements were taken at rest and during continuous adenosine infusion (70/140/210 mcg/kg/min). Initial meanPAvel’s (rest and hyperemic) were correlated with validated surrogate prognostic parameters (CMR: RV ejection fraction, RVEF; RV end systolic volume indexed, RVESVI; RHC: PVRI, CI; biomarker: NT-pro brain natriuretic peptide, NTpBNP; clinical: 6-min walk distance, 6MWD), a measure of pulmonary arterial stiffness (elastic modulus) and volumetric estimation of RV ventriculoarterial (VA) coupling. Changes in meanPAvel’s were correlated with changes in comparator parameters over time.

At initial assessment, meanPAvel at rest correlated significantly with PVRI (inversely), CI (positively) and elastic modulus (inversely) (*R* ^2^ > 0.37,*P* < 0.05 for all), whereas meanPAvel at peak hyperemia correlated significantly with PVRI, RVEF, RVESVI, 6MWD, elastic modulus and VA coupling (*R* ^2^ > 0.30,*P* < 0.05 for all). Neither resting or hyperemia-derived meanPAvel correlated with NTpBNP levels. Initial meanPAvel at rest correlated significantly with RVEF, RVESVI, CI and VA coupling at follow up assessment (*R* ^2^ > 0.2,*P* < 0.05 for all) and initial meanPAvel at peak hyperemia correlated with RVEF, RVESVI, PVRI and VA coupling (*R* ^2^ > 0.37,*P* < 0.05 for all). Change in meanPAvel at rest over time did not show statistically significant correlation with change in prognostic parameters, while change in meanPAvel at peak hyperemia did show a significant relationship with ΔRVEF, ΔRVESVI, ΔNTpBNP and ΔCI (*R* ^2^ > 0.24,*P* < 0.05 for all).

**Conclusion:**

MeanPAvel during peak hyperemia correlated with invasive, non-invasive and clinical prognostic parameters at different time points. Further studies with predefined clinical endpoints are required to evaluated if this novel tool is a marker of disease progression in patients with pulmonary vascular disease.

## Background

Pulmonary arterial hypertension (PAH) is characterised by a pathological increase in the resistance of the pulmonary circulation secondary to arterial wall remodelling, vasoconstriction and in situ thrombosis (pulmonary vascular disease, PVD). Progressive obliteration of the pulmonary vascular bed eventually leads to right ventricular (RV) failure and premature death. Therapeutic advances have contributed to better long-term outcomes but disease progression remains difficult to predict and objectively monitor necessitating new methods.

We previously demonstrated proof-of-concept for a novel non-invasive method to assess ‘cardiopulmonary reserve’ as it pertains to PAH by measuring the average pulmonary arterial blood flow velocity (meanPAvel) at rest and during standardised intravenous (IV) adenosine infusion using phase-contrast cardiovascular magnetic resonance (CMR) [1]. This approach to ‘stressing’ the cardiopulmonary unit, whilst novel, was feasible, safe and simple, with the results confirming that meanPAvel at peak hyperemia was an excellent functional correlate for cardiopulmonary reserve across a range of clinical risk phenotypes.

As a marker of cardiopulmonary reserve and with the advantage of ameliorating the impact of unrelated systemic processes on variables measured at rest, we hypothesised that meanPAvel at peak hyperemia may provide prognostic information at initial assessment and during follow up. To evaluate this, we investigated the association of meanPAvel at rest and during peak hyperaemia with accepted RHC-derived, CMR-derived, biomarker and clinical prognostic surrogate markers in a cohort of patients with confirmed PAH or at high risk for incident PAH. Specifically, we hypothesised that relative to meanPAvel at rest, meanPAvel at peak hyperaemia at initial assessment would be more closely associated with comparator parameters at initial and follow-up assessments, and that changes in meanPAvel at peak hyperemia would correlate more closely with changes in these prognostic markers over time.

## Methods

We prospectively recruited participants with known or suspected PAH and a clinical indication for a right heart catheter (RHC) through a single tertiary-referral centre over a 14-month period (2013–2014). Patients were ineligible if they were <18 years old; pregnant; had known or suspected PAH due to congenital heart disease with left-to-right shunt, or portopulmonary hypertension; or, had a contraindication to CMR or IV adenosine. Right heart catheterization for suspected PAH was deemed clinically indicated by pulmonary hypertension experts following a comprehensive review of clinical status and ancillary investigations, in accordance with guidelines [2]. All participants provided written informed consent and protocols were approved by the Local Institutional Research Ethics Committee.

At initial assessment, all participants underwent a RHC and CMR with IV adenosine within 48 h, with PAH confirmed by hemodynamic criteria (mean pulmonary arterial pressure, mPAP, >25 mmHg; mean pulmonary arterial wedge pressure, mPAWP, <15 mmHg; and pulmonary vascular resistance, PVR >3 Woods units, WU). Participants with mPAWP > 15 mmHg were excluded and did not undergo vasoreactive testing. Patients with PAH were invited to undergo repeat RHC and CMR with vasoreactivity testing and participants with suspected but excluded PAH (high-risk for incident PAH) invited to undergo repeat CMR with vasoreative testing approximately 6 months after their initial study. Between assessments, patients were administered guideline-appropriate management.

Right heart catheterization was performed via the right femoral vein with a 7 F Swan-Ganz thermodilution pulmonary artery catheter (Edwards Lifesciences, California, USA). Systemic arterial pressures were monitored by arm cuff. Complete resting hemodynamic assessment was performed in accordance with current guidelines. Intravenous (IV) adenosine (Adenoscan, 3 mg/mL, Astellas Pharma, Tokyo, Japan) was infused via peripheral vein at three increasing doses: 70-, 140-, and 210mcg/kg/min, and repeat hemodynamic assessment (mPAP/mPAWP/CI/PVR) conducted after a minimum of two minutes’ infusion at each dose.

**Fig. 1:**
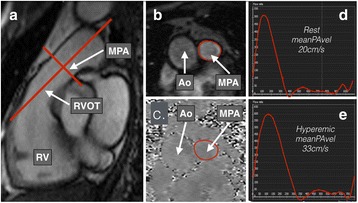
Reference sequences for main pulmonary arterial (MPA) phase contrast imaging were two double-oblique orthogonal views along the main axis of the pulmonary trunk (**a**). The endocardial border of the MPA was manually outlined at all 20 reconstructed cardiac phases (**b** and **c**) permitting flow velocity profiles to be generated at rest and hyperemia (**d** and **e** respectively: flow velocity in ml/s on *y* axis and time in milliseconds on *x* axis). MeanPAvel was calculated as the average blood flow velocity across all cardiac phases. Ao = aorta, RV = right ventricle, RVOT = right ventricular outflow tract

CMR was performed with a 1.5 T magnet (Magnetom, Siemens, Erlangen, Germany) with images obtained at end-expiration. Volumetric and functional analyses were performed using steady-state free procession (SSFP) sequences of the atria and ventricles. Flow imaging through the main pulmonary artery was performed 1.5-2 cm above the pulmonary valve using a velocity-encoded gradient echo sequence with an upper velocity limit of 150 cm/s, temporal resolution of 39 ms and spatial resolution of 1.8x1.8x6mm (Fig. 1). Adenosine was infused using the same protocol as during RHC, with flow imaging through the pulmonary trunk repeated after a minimum of 2 min at each adenosine dose. Analyses were performed offline using specialized software (CMR42, Circle Cardiovascular Imaging Inc., Calgary, Canada). Ventricular endocardial and epicardial contours were manually outlined at end-diastole and end-systole to permit calculation of ventricular volumes and myocardial mass by Simpson’s method. Blood flow through the pulmonary trunk was measured by outlining the endovascular border at all 20 reconstructed cardiac phases permitting calculation of mean pulmonary arterial blood flow velocity (meanPAvel), elastic modulus (pulse pressure x minA/(maxA-minA)) and a volumetric estimation of ventriculoarterial (VA) coupling (RVESV/RV stroke volume) [3]. All participants underwent 6-min walk tests and had blood samples taken for NT-proBNP quantification, which was centrifuged within 1 h for 10 min at 2800 rpm, and stored at −80 °C until analysis using an ELISA kit (USCN Life Science, Texas, USA).

### Treatment Strategy

The objective was to assess the relative strength of association of meanPAvel measured at rest and at peak hyperemia with comparator surrogate prognostic parameters. Treatment of participants with PAH was at the discretion of treating physicians and in line with current guidelines [4].

### Comparator Surrogate Prognostic Parameters

Comparator surrogate prognostic parameters for initial and serial analyses were defined *a priori* as: 1) CMR-derived: right ventricular ejection fraction (RVEF) and right ventricular end systolic volume index (RVESVI); 2) Biomarker: NT-proBNP levels; 3) RHC-derived: pulmonary vascular resistance index (PVRI) and cardiac index (CI); 4) Clinical: six-minute walk distance (6MWD). In addition to these validated serial prognostic parameters, two other physiologically-relevant parameters were assessed: a marker of pulmonary arterial stiffness (elastic modulus; at initial assessment only), and volumetric estimation of VA coupling (at initial and follow-up assessments).

### Statistical Analysis

Normally distributed continuous variables were expressed as means ± standard deviations. Normality was assessed using the Shapiro-Wilk normality test. Scatterplots of univariate associations were reviewed and, where appropriate, Pearson correlation coefficients were used to explore the strength of linear relationships between meanPAvel at rest and during hyperemia with comparator prognostic parameters and Spearman correlation coefficients were used to explore the strength of relationships that were curvilinear and where there was a significant departure from a Gaussian distribution. More specifically, meanPAvel measured at rest and during hyperemia at the initial assessment were correlated with initial and follow up comparator parameters; and changes in meanPAvel at rest and during hyperemia were correlated with changes in comparator parameters between initial assessment and follow up. Statistical analyses were performed using a software package (GraphPad Prism version 6.0, San Diego, California, USA), with a two-sided *P* value of <0.05 considered statistically significant.

## Results

Thirty-one participants were recruited (PAH *n* = 19; suspected but excluded PAH: ‘High Risk’ *n* = 12) of which 5 were ineligible (mPAWP > 15 mmHg *n* = 4; claustrophobia *n* = 1). 20 underwent repeat investigations (PAH *n* = 14; High Risk *n* = 6) after a median period of 8 months and 21 days (loss to follow-up in PAH group: death from RV failure *n* = 1; geographical relocation *n* = 1; declined *n* = 1, and High Risk group: geographical relocation *n* = 2; intercurrent illness *n* = 1) and were included in the present study. Ten of the 14 PAH participants had both repeat CMR and RHC and 4 had CMR only (declined repeat RHC *n* = 4). Demographic and clinical characteristics of participants are presented in Table 1, with suspected but excluded PAH participants considered high risk for incident PAH on the basis of the high prevalence of connective tissue disease, unaccounted for exertional breathlessness with disproportionately reduced lung diffusing capacity, elevated NTpBNP levels in the absence of left ventricular myocardial disease or renal dysfunction, elevated estimated pulmonary arterial systolic pressure by transthoracic echocardiography >40 mmHg (*n* = 6) and ‘borderline’ abnormal resting hemodynamics (*n* = 2 with rest mPAP of 21-24 mmHg) [5].

**Table 1:** Demographic and clinical characteristics of participants

	PAH	High risk	*P* value
Participants, *n*	14	6	
Female (%)	11 (79)	5 (83)	0.98
Age, years	54 ± 14	63 ± 7.6	0.18
BSA, m^2^	1.79 ± 0.25	1.83 ± 0.17	0.79
PAH aetiology (%)			
Idiopathic	10 (71)		
CTD-associated	4 (29)		
Coexisting conditions (%)			
Diabetes mellitus	2 (14)	0 (0)	<0.05
Smoker	1 (12)	0 (0)	<0.05
CTD	5 (36)	5 (83)	<0.05
NYHA functional class (%)			
II	3 (21)	6 (100)	<0.05
III	11 (79)	0 (0)	<0.05
Therapy, initial/follow-up (%)			
Phosphodiesterase-5 inhibitor	1 (7)/0 (0)		
Combination	13 (93)/14 (100)		
Creatinine, μmol/L	84 ± 21	83 ± 34	0.96
D_L_CO, %	55 ± 15	58 ± 19	0.76
%FVC/%D_L_CO	1.41 ± 0.41	1.38 ± 0.27	0.92

Mean absolute values of comparator, investigational and other relevant parameters, and their relative changes over time, are summarised in Table 2.

**Table 2:** Absolute values and relative change in comparator (bold text) and investigational parameters (mean ± SD)

	Parameter	Group	Initial	Follow-up	Change (%)
CMR-derived	**RVEF (%)**	**PAH**	**41 ± 17**	**43 ± 13**	**18 ± 47**
		**High risk**	**60 ± 7**	**59 ± 6**	**−1 ± 13**
	**RVESVI** (**ml/m** ^**2**^)	**PAH**	**50 ± 26**	**54 ± 25**	**17 ± 49**
		**High risk**	**24 ± 7**	**25 ± 8**	**4 ± 19**
	**meanPAvel at rest** (**cm/s**)	**PAH**	**9.7 ± 2.3**	**10.0 ± 3.0**	**5 ± 27**
		**High risk**	**14.4 ± 2.6**	**13.9 ± 3.6**	**−4 ± 23**
	**meanPAvel during hyperemia** (**cm/s**)	**PAH**	**12.2 ± 3.3**	**12.9 ± 6.3**	**6 ± 32**
		**High risk**	**21.5 ± 4.3**	**19.8 ± 4.9**	**−8 ± 8**
	RVEDVI (ml/m^2^)	PAH	84 ± 19	91 ± 22	8 ± 15
		High risk	61 ± 14	61 ± 17	0 ± 13
	LVEF (%)	PAH	65 ± 11	68 ± 9	5 ± 21
		High risk	69 ± 4	66 ± 6	−3 ± 9
RHC-derived	**CI** (**L/min/m** ^**2**^)	**PAH**	**2.35 ± 0.76**	**2.59 ± 0.81**	**13 ± 19**
		**High risk**	**2.80 ± 0.55**	**n/a**	**n/a**
	**PVRI** (**WU/m** ^**2**^)	**PAH**	**5.5 ± 3.2**	**5.3 ± 3.4**	**11 ± 47**
		**High risk**	**1.0 ± 0.4**	**n/a**	**n/a**
	mPAP (mmHg)	PAH	46 ± 16	48 ± 14	9 ± 24
		High risk	19 ± 4	n/a	n/a
	mPAWP (mmHg)	PAH	10 ± 2	8 ± 5	3 ± 30
		High risk	10 ± 3	n/a	n/a
Clinical/Biomarker	**NTpBNP** (**pg/ml**)	**PAH**	**2310 ± 1354**	**2740 ± 1361**	**71 ± 200**
		**High risk**	**1345 ± 718**	**1289 ± 693**	**−3 ± 13**
	**6MWD** (**m**)	**PAH**	**421 ± 81**	**435 ± 62**	**7 ± 18**
		**High risk**	**484 ± 71**	**437 ± 94**	**−3 ± 8**

### Response to adenosine

**Fig. 2:**
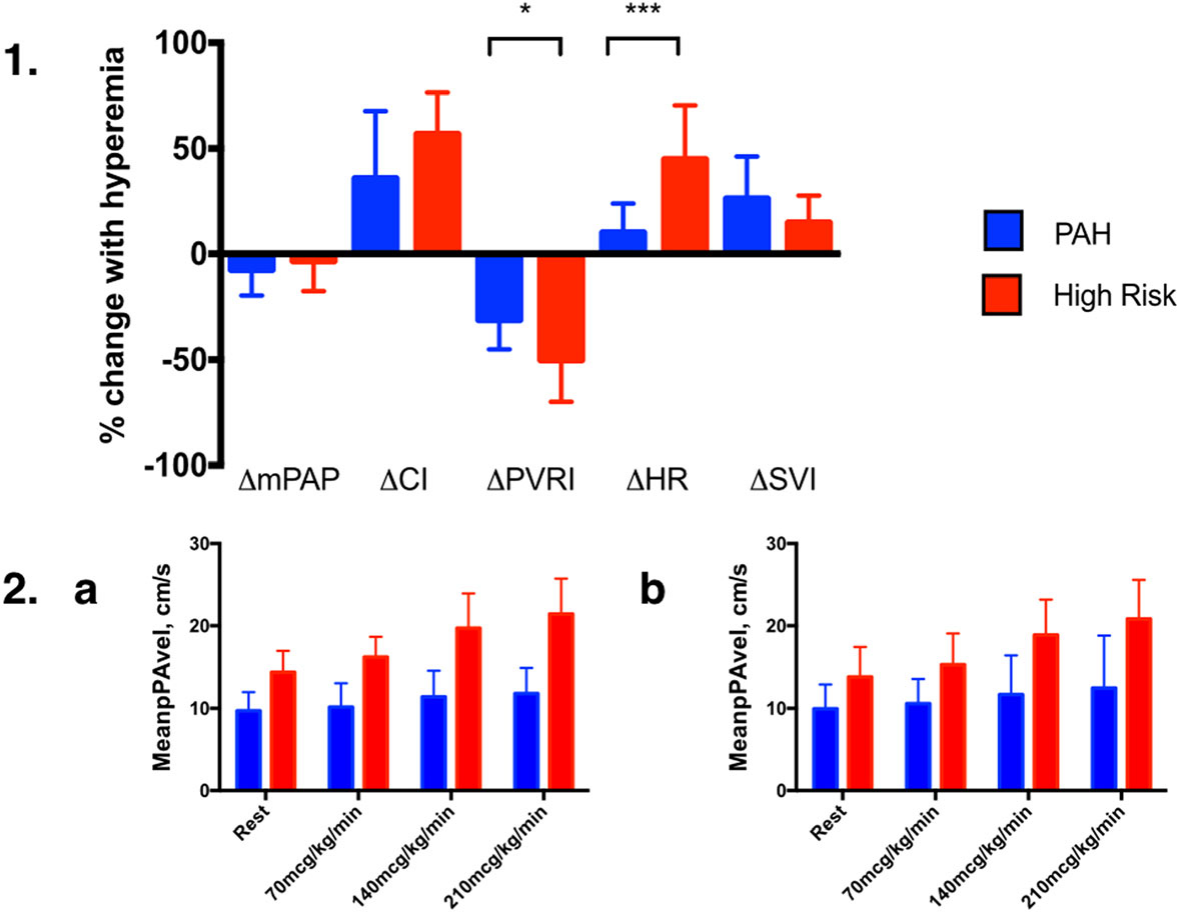
Changes in RHC-derived hemodynamics (panel 1) and CMR-derived meanPAvel (initial assessment, panel 2a; follow-up assessment, panel 2b) in response to adenosine in PAH and High Risk participants. At RHC, intravenous adenosine produced a dose dependent reduction in PVRI driven predominantly by a higher CI rather than a lower mPAP or transpulmonary gradient (not illustrated). At CMR, these changes were reflected by a dose-dependent rise in meanPAvel. Changes were more pronounced in High Risk participants relative to those with PAH

The hemodynamic and meanPAvel changes in response to adenosine are shown in Figs. 1 and 2. Systemic blood pressure (SBP) did not change significantly during adenosine (systolic SBP −6 ± 13 and 0 ± 18% for PAH and High Risk groups respectively, *P* = 0.38 and 0.23 respectively – not displayed graphically). There was minimal intra-observer and interobserver variability for meanPAvel measures (mean bias: −0.01 and −0.0000238; 95% limits of agreement: −0.21–0.19 and −0.27–0.27 respectively) at all doses.

### Correlation between mean pulmonary arterial blood flow velocities and prognostic parameters measured at initial assessment

**Fig. 3:**
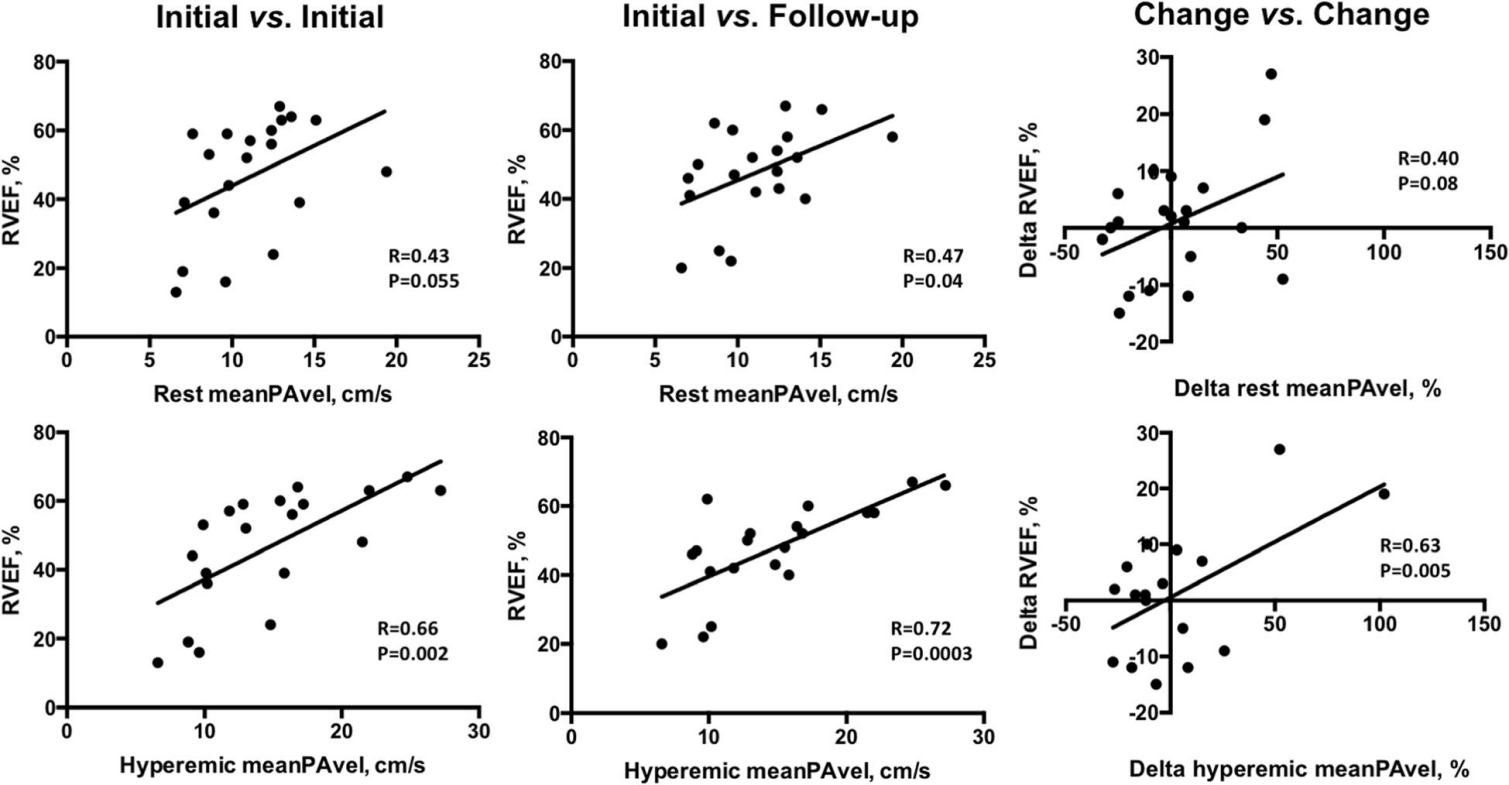
Scatterplots illustrating the relationship between RVEF, meanPAvel at rest and meanPAvel at hyperemia at different time points *R* = Pearson correlation coefficient.

Correlation coefficients are presented in Table 3. MeanPAvel at rest showed strong positive correlation with CI and very strong negative correlation (curvilinear) with PVRI. There was moderate but statistically insignificant correlation with CMR indices (negative with RVESVI and positive with RVEF, Fig. 3) and 6MWD (positive), but no correlation with NTpBNP. Elastic modulus, a measure of the pressure change required to drive a relative change in the PA lumen size, showed strong negative correlation with meanPAvel at rest. Volumetric estimation of ventriculoarterial coupling showed moderate strength negative correlation with meanPAvel at rest. Therefore a high effective arterial elastance:maximal systolic elastance ratio (estimated here by CMR-derived RVESV/RVSV), suggestive of RV-pulmonary uncoupling, was associated with lower meanPAvels. Relative to meanPAvel at rest, meanPAvel at hyperemia correlated more closely with all parameters except RHC-derived CI at rest and NTpBNP.

**Table 3:** Correlation coefficients investigating the relationship between meanPAvel and comparator parameters measured during initial assessment

Comparator parameter	meanPAvel at rest	*P* value	meanPAvel during hyperemia	*P* value
RVEF	0.43 [−0.01 to 0.74]	0.055	0.66** [0.31 to 0.85]	0.002
RVESVI	−0.38 [−0.71 to 0.09]	0.10	−0.70*** [−0.88 to −0.36]	0.0006
NTpBNP	−0.19 [−0.61 to 0.30]	0.44	−0.21 [−0.62 to 0.29]	0.40
PVRI	−0.81**** [−0.93 to −0.57]	<0.0001	−0.88*** [−0.95 to −0.70]	<0.0001
CI	0.65** [0.30 to 0.85]	0.002	0.41 [−0.03 to 0.72]	0.07
6MWD	0.43 [−0.04 to 0.74]	0.07	0.58** [0.17 to 0.82]	0.009
Elastic modulus	−0.61** [−0.83 to −0.23]	0.004	−0.65** [−0.85 to −0.29]	0.001
VA coupling	−0.49* [−0.77 to −0.05]	0.03	−0.75*** [−0.90 to −0.46]	0.0001
Maximum CI	0.60** [0.22 to 0.83]	0.005	0.65** [0.29 to 0.85]	0.002
Minimum PVRI	−0.76** [−0.90 to −0.47]	0.001	−0.74*** [−0.94 to −0.62]	0.0002

[95% confidence interval]

*P < 0.05

**P < 0.01

***P < 0.001

****P < 0.0001

Given the weaker association between CI and meanPAvel at peak hyperemia, compared to meanPAvel at rest, we assessed the relationship between meanPAvel’s and hemodynamic measurements made during hyperemia. In this regard, meanPAvel at rest and during hyperemia correlated closely with the maximum CI (*R* = 0.60, *P* = 0.005 and *R* = 0.65, *P* = 0.002 respectively) and minimum PVRI (*R* = −0.67, *P* = 0.001 and *R* = −0.74, *P* = 0.0002 respectively) during adenosine challenge. This is consistent with earlier work demonstrating close correlation between meanPAvel and invasively-derived hemodynamics at rest and during adenosine infusion [1].

### Correlation between initial mean pulmonary arterial blood flow velocities and follow up prognostic parameters

MeanPAvel measured at rest showed moderate strength statistically significant correlation with RVEF (Fig. 3), RVESVI and VA coupling at follow up. Strong negative correlation with follow up PVRI and positive correlation with follow up CI were found. In contrast, there was weak and statistically insignificant correlation with follow up NTpBNP levels and no correlation with 6MWD. MeanPAvel measured during peak hyperemia correlated more closely with all parameters except resting CI (R = 0.67 *vs.* 0.59 for meanPAvel at rest *vs.* peak hyperemia), 6MWD and NTpBNP. Correlation coefficients are presented in Table 4.

**Table 4:** Correlation coefficients investigating the relationship between meanPAvel measured during initial assessment with follow-up comparator parameters

Comparator parameter	meanPAvel at rest	*P* value	meanPAvel during hyperemia	*P* value
RVEF	0.47* [0.04 to 0.76]	0.04	0.72*** [0.41 to 0.88]	0.0003
RVESVI	−0.53* [−0.79 to −0.10]	0.02	−0.71*** [−0.88 to −0.37]	0.0005
NTpBNP	−0.40 [−0.73 to 0.06]	0.09	−0.42 [−0.73 to 0.04]	0.07
PVRI	−0.62* [−0.89 to −0.01]	0.05	−0.70* [−0.92 to −0.15]	0.02
CI	0.67* [0.11 to 0.90]	0.03	0.59 [−0.02 to 0.88]	0.06
6MWD	0.23 [−0.26 to 0.63]	0.34	0.10 [−0.39 to 0.54]	0.71
VA coupling	−0.46* [−0.75 to −0.01]	0.04	−0.71*** [−0.88 to −0.37]	0.0005

[95% confidence interval]

*P < 0.05

**P < 0.01

***P < 0.001

### Correlation between changes in mean pulmonary arterial blood flow velocities and changes in comparator prognostic parameters

There were no statistically significant associations between change in meanPAvel measured at rest and change in comparator prognostic parameters, although statistical power was reduced with regard to RHC-derived parameters owing to fewer follow-up invasive procedures (*n* = 14 *vs. n* = 10). In contrast, change in meanPAvel measured at hyperemia correlated moderate-strongly with all comparator prognostic parameters except 6MWD, PVRI and VA coupling (*R* = 0.30, *P* = 0.30/R = −0.39, *P* = 0.27/R = −0.44, *P* = 0.06 respectively). Similar trends were seen when only confirmed PAH participants were analysed. Correlation coefficients are presented in Table 5.

**Table 5:** Correlation coefficients investigating the relationship between changes in meanPAvel and changes in comparator parameters over time

Comparator parameter	meanPAvel at rest	*P* value	meanPAvel during hyperemia	*P* value
ΔRVEF	0.40 [−0.05 to 0.72]	0.08	0.63** [0.23 to 0.85]	0.005
ΔRVESVI	−0.23 [−0.61 to 0.24]	0.34	−0.50* [−0.78 to −0.05]	0.03
ΔNTpBNP	−0.30 [−0.71 to 0.20]	0.26	−0.52* [−0.82 to −0.01]	0.04
ΔPVRI	−0.07 [−0.64 to 0.55]	0.84	−0.39 [−0.82 to 0.32]	0.27
ΔCI	0.50 [−0.14 to 0.85]	0.12	0.62* [−0.02 to 0.90]	0.05
Δ6MWD	0.26 [−0.25 to 0.66]	0.31	0.30 [−0.27 to 0.72]	0.30
ΔVA coupling	−0.28 [−0.64 to 0.18]	0.23	−0.44 [−0.74 to 0.02]	0.06

[95% confidence interval]

*P < 0.05

**P < 0.01

Despite its routine use, 6MWD has many limitations as a serial prognostic marker (*e.g.* day-to-day variation, learning effect and comorbidities), with registry data showing a decline in 6MWD confers a worse prognosis but a rise in 6MWD has no impact on survival [6]. In those participants with a decline in 6MWD (*N* = 9) in the present study, correlation with change in meanPAvel at hyperemia improved but statistical significance was not met (*R* = −0.52, *P* = 0.22) possibly due to the low sample size.

## Discussion

Blood flow velocity through the main pulmonary artery is thought to decline in PAH due to impeded passage of cardiac output through remodelled microcirculation, dilation of proximal pulmonary vessels and eventually, impaired RV function [7]. Not surprisingly therefore, mean pulmonary arterial blood flow velocity (meanPAvel) measured at rest has been shown to correlate with measures of RV function, arterial load and exercise capacity, which are all important determinants of prognosis in PAH [7–11]. This study adds to our knowledge by demonstrating correlation between our non-invasive measure of ‘cardiopulmonary’ reserve (meanPAvel at peak hyperemia, [1]) and validated invasively and non-invasively derived surrogate prognostic markers at baseline and six month follow-up. This marker was superior to meanPAvel when measured at rest. This data would support further studies being undertaken with hard clinical endpoints, to establish if this novel marker would provide incremental prognostic information in patients with PAH or at high risk for incident PAH.

PAH is principally defined by the plexogenic pulmonary vasculopathy that underlies its genesis, increasing RV afterload and stress. These two compartments (the RV and pulmonary vasculature) are integrally related and not surprisingly therefore, measures of RV afterload (static *e.g.* PVR, and oscillatory *e.g.* pulmonary arterial stiffness, components), stress (*e.g.* NTpBNP), and functional adaptation (*e.g.* RVEF, RVESVI) all provide independent prognostic information. In isolation however, each parameter tends to provide a limited picture of the state of the ‘cardiopulmonary’ unit as a whole. In our study, meanPAvel at peak hyperemia correlated significantly with measures of RV afterload (*e.g.* PVRI and elastic modulus), RV functional adaptation (*e.g.* RVEF/RVESVI) and VA coupling (defined as the ratio of maximal systolic RV elastance: effective arterial elastance), suggesting a capacity to simultaneously interrogate the pulmonary vasculature, the RV, and their interaction in a simple, non-invasive fashion. The capacity to assess the RV and pulmonary vasculature as a collective unit may translate to improved ability to discriminate changes in ventricular function, arterial load, or both when compared to, for example, RVEF, a serial CMR measure that conveys consistent prognostic information in PAH patients [3, 12, 13].

‘Traditional’ prognostic parameters are generally measured at rest and are subject to the influence of unrelated systemic factors such as anxiety, hypertension and sedation which may be compounded by serial assessment. Moreover, they lack the capacity to assess physiological reserve, that is, they provide a static measure of a dynamic disease process. Our protocol, like other physiological stress tests, negates the influences of unrelated systemic factors whilst permitting assessment of reserve as it pertains to PAH, that is, the degree of functional pulmonary circulation recruitable by endothelial independent mechanisms with IV adenosine (‘vascular reserve’) and the capacity of the RV to increase blood flow in response to downstream circulatory recruitment (‘RV reserve’: chronotropic/inotropic). We postulate that the generally closer association found between meanPAvel at peak hyperemia with validated prognostic parameters, compared with meanPAvel measured at rest, was due to the capacity to assess the cardiopulmonary unit as whole and its’ reserve while ameliorating the impact of unrelated systemic processes.

Acknowledging the above, not all validated prognostic comparators correlated significantly with meanPAvel at peak hyperemia. Most notably, NTpBNP levels measured at initial assessment did not correlate with meanPAvel measured at the same assessment or at follow-up, although the relative change in NTpBNP correlated with the change in meanPAVel at peak hyperemia over time. Previous studies have demonstrated association between baseline NTpBNP levels, resting hemodynamics, lung function, peak VO2 and prognosis, CMR-derived measures of RV function, and association between change in NTpBNP over time and survival [14–19]. The discordant findings of strong correlation between meanPAVel at peak hyperemia, resting hemodynamics and markers of RV functional adaptation (RVEF, RVESVI) but no correlation with NTpBNP levels in absolute terms were unexpected and difficult to explain biologically: they may therefore reflect a statistical anomaly. A lack of correlation between change in meanPAvel and change in PVRI over time was likely due to the small sample size undergoing repeat RHC (*N* = 10) or, as earlier discussed, the impact of unrelated systemic factors on resting measures that may compound with serial assessment. No correlation between change in meanPAvel at peak hyperemia and change in 6MWD over time may reflect the inherent limitations of serial 6MWDs and is in keeping with registry data showing a decline in 6MWD is prognostically relevant whereas an increase is not [6]. Closer association between these variables in the subset of patients that experienced a decline in 6MWD (*n* = 9: *R* = 0.59, *P* = 0.22) is in keeping with our other findings, acknowledging that statistical significance was not met, likely due to a small sample size.

### Study limitations

The major limitations are the small sample size, single centre study design, and lack of clinical outcome data. Findings from this study should be considered hypothesis generating. Repeat vasoreactivity testing via invasive or non-invasive protocols is novel, with this study designed to explore its potential clinical utility by assessing relative strength of association across a range of validated prognostic parameters. Whether meanPAvel provides independent prognostic information cannot be determined. Evidence for potential clinical utility of repeat standardised vasoreactivity testing using this protocol is provided by the combination of physiologically appropriate correlation across a range of relevant CMR-derived, RHC-derived, biomarker and clinical variables; and correlation of generally greater strength at hyperemia compared to rest. Larger studies are required to confirm these findings.

## Conclusion

Mean pulmonary arterial blood flow velocity measured using CMR during non-invasive standardised pulmonary vasoreactive testing correlated moderate-strongly with a range of CMR-derived, RHC-derived, biomarker and clinical prognostic variables at initial assessment and at follow up. Change in meanPAvel at peak hyperemia on repeat vasoreactivity testing correlated with change in these variables over time, although correlation was generally weaker. Compared with meanPAvel measured at rest, correlation was generally stronger with meanPAvel measured at peak hyperemia. The novel parameter of meanPAvel at peak hyperemia may provide prognostic information by simultaneously interrogating the pulmonary vasculature and RV functional capacity whilst ameliorating the impact of unrelated systemic processes, although larger trials are required to confirm these findings.

## Abbreviations

6MWD: 6-munute walk distance
CI: Cardiac index
CMR: Cardiovascular magnetic resonance
meanPAvel: Mean pulmonary arterial blood flow velocity
mPAP: Mean pulmonary arterial pressure
NTpBNP: N-terminal pro-BNP
PVD: Pulmonary vascular disease
PVRI: Pulmonary vascular resistance indexed
RHC: Right heart catheter
RVEF: Right ventricular ejection fraction
RVESVI: Right ventricular end systolic volume indexed
